# Protein–Polysaccharide Composite Materials: Fabrication and Applications

**DOI:** 10.3390/polym12020464

**Published:** 2020-02-17

**Authors:** Elizabeth J. Bealer, Shola Onissema-Karimu, Ashley Rivera-Galletti, Maura Francis, Jason Wilkowski, David Salas-de la Cruz, Xiao Hu

**Affiliations:** 1 Department of Physics and Astronomy, Rowan University, Glassboro, NJ 08028, USA; bealere0@students.rowan.edu (E.J.B.); riveragaa4@students.rowan.edu (A.R.-G.); 2 Department of Biomedical Engineering, Rowan University, Glassboro, NJ 08028, USA; onissemas7@students.rowan.edu (S.O.-K.); francism4@students.rowan.edu (M.F.); wilkowskj8@students.rowan.edu (J.W.); 3 Department of Chemistry and Biochemistry, Rowan University, Glassboro, NJ 08028, USA; 4 Department of Chemistry, Rutgers University, Camden, NJ 08102, USA; ds1191@camden.rutgers.edu; 5 Center for Computational and Integrative Biology, Rutgers University, Camden, NJ 08102, USA; 6 Department of Molecular and Cellular Biosciences, Rowan University, Glassboro, NJ 08028, USA

**Keywords:** protein and polysaccharide, composite material, tissue regeneration, drug delivery and nanomedicine, health and nutrition, water treatment

## Abstract

Protein–polysaccharide composites have been known to show a wide range of applications in biomedical and green chemical fields. These composites have been fabricated into a variety of forms, such as films, fibers, particles, and gels, dependent upon their specific applications. Post treatments of these composites, such as enhancing chemical and physical changes, have been shown to favorably alter their structure and properties, allowing for specificity of medical treatments. Protein–polysaccharide composite materials introduce many opportunities to improve biological functions and contemporary technological functions. Current applications involving the replication of artificial tissues in tissue regeneration, wound therapy, effective drug delivery systems, and food colloids have benefited from protein–polysaccharide composite materials. Although there is limited research on the development of protein–polysaccharide composites, studies have proven their effectiveness and advantages amongst multiple fields. This review aims to provide insight on the elements of protein–polysaccharide complexes, how they are formed, and how they can be applied in modern material science and engineering.

## 1. Introduction

The use of composites as biomaterials has been extremely effective in tissue engineering, drug delivery, and the food industry. Generally, a composite material can be composed of polymers, proteins, ceramics, or polysaccharides [1]. Composites can be created with a wide variety of materials that range in texture, composition, and size. Metal and carbon nanoparticles can also be added and exhibit the unique properties of each material being used [2]. This inherent versatility offers a greater alternative to synthetic polymers alone [2]. In this review, specific research on various protein–polysaccharide composite combinations will be covered.

Protein–polysaccharide composites play a large role in current research in biomedical applications, such as wound healing, electrical devices, and nanomedicine [2]. The integration of protein–polysaccharide composites in forming hydrogels to fill cartilage defects [3], electrospinning to create antimicrobial properties for wound repair [4], and generating films for use in food packaging and drug deliveries [5] has led to improvements in these processes. Protein materials commonly used in composites include silk, keratin, soy, collagen, gelatin, resilin, corn zein, and wheat gluten. Each protein can be distinguished by its mechanical, chemical, electrical, and optical properties, which allow for a broad range of applications [5,6]. Proteins are synthesized in template-directed polymerization to produce monodispersed linear polymers composed of distinct monomers or residues. In complex tissues, a wide combinational range of amino acid monomers are used for synthesis. These monomers are linked through amide bonds where only L-amino acids are used. The sequence of amino acids is known as the primary structure of proteins, whereas secondary, tertiary, and quaternary structures undergo the process of folding in order to reach its ‘native’ conformation [7]. Common secondary structures, such as α-helices, β-sheets, and β-turns, in proteins are where π–π interactions between aromatic amino acids and hydrogen bonding between amide bonds occur.

Through controlled settings, protein materials demonstrate the capability of responding to numerous stimuli, such as temperature, electrical, magnetic, and enzymatic stimuli [8]. Material constructs can originate from a variety of proteins due to their ability to offer sites of attachment at the side chains. These connections could include drugs, crosslinking agents, or pendant groups that can manipulate the mechanical and chemical properties of a material [9]. In addition, multifunctional composite materials have been created through the fabrication of protein hybrids. Materials possessing an array of functions and mechanical properties for specific tissues are created using recombinant polypeptides with the help of proteins, such as elastin and collagen [10]. However, this technique is limited due to the high cost of the technology needed for this fabrication method. Certain proteins are limited in their cell biocompatibility or range of mechanical properties [6]. Nevertheless, protein-based materials have beneficial properties in the stability of drug attachments, biodegradability, and biocompatibility. The aforementioned reasons increase proteins’ favorability for use in composites [11,12,13,14,15].

Polysaccharides are constructed from monomeric sugars that are linked together by O-glycosidic linkages and have the ability to store material, compose structural components, and act as protective materials [16,17,18]. Typical polysaccharides include starch, cellulose, pectin, alginates, chitosan, and hyaluronic acid found in plants, algae, or animals [19,20]. With polysaccharide materials being so abundant, they are inexpensive and readily available biopolymers. Polysaccharides have a number of advantages over nucleic acids and proteins for applications of material science since they are generally more stable, and usually do not denature on heating [20]. The wide diversity of polysaccharides yields materials with low, intermediate, and high molecular weights and varying polydispersity indexes. The varying polydispersity is accounted for by the changes between polysaccharides, including their structures, solubility, and toxicity [2].

The extensive use of polysaccharides is highly favored due to their biocompatibility, biodegradability, high chemical reactivity, and polyfunctionality [21]. The innate properties and various structural changes grant molecular and biological advantages for use in the preparation of nanomaterials and nanocomposites. Polysaccharides are hydrophilic in nature, which also provides an advantage in creating the polysaccharide–protein complex because it acts as a stabilizing agent [22].

Polysaccharide–protein composites have become increasingly popular for use in the biomedical field to form scaffolds, particles, films, fibers, and gels because of their intermolecular interactions with their matrices [23,24,25,26]. These complexes interact by creating strong bonds with each other through hydrophobic–hydrophobic interactions as well as electrostatic interactions [22]. The formation of these composites allows for the material properties of the protein to be strengthened through the blend of the polysaccharide [23]. The composites have the ability to take on the properties of the materials present, such as their size [2,23]. Overall, the creation of a protein–polysaccharide complex can be manipulated into displaying the properties desired, which can enhance the biodegradability, biocompatibility, and mechanical properties [27].

These biopolymer composites may be used to fabricate structures on the nanometer or micrometer scales. The composite particles that are formed can be used for the protection or delivery of a pharmaceutical or nutrient, such as a drug or bioactive lipid [28,29]. In addition, biocomposites from proteins and polysaccharides may be used to create particulates to replace fat in certain products through various types of biopolymer–biopolymer associative interactions [29]. Therefore, it is important to design and fabricate appropriate polysaccharide–protein biopolymers with specific compositions and structures depending on the intent of use. The selection of particular materials to form biocomposites depends on a number of factors: The ability to assemble composites, the functional attributes of the composites formed (such as their size, shape, charge, and stability), and the cost, ease of use, and legal status of the ingredients used.

**Figure 1:**
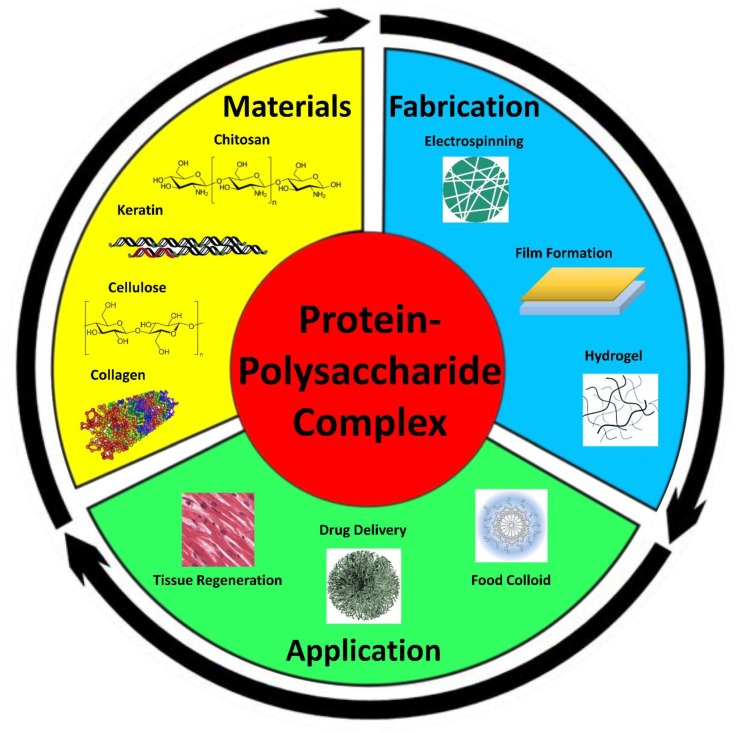
Protein–polysaccharide composite materials can be fabricated from a variety of protein and polysaccharide sources. These complex materials can then be processed into variable shapes with unique properties for a multitude of applications.

The purpose of this review is to provide an in-depth analysis on the structural integrity, fabrication, and application of protein–polysaccharide-based compounds (Figure 1). An overview of various protein materials, such as silk, keratin, and collagen, along with a combination of polysaccharide materials, such as cellulose, pectin, and chitosan, will be covered to provide a fundamental examination on a molecular level of these biopolymers. By examining each component of the composite materials, a further understanding of application and fabrication can be established as well as bringing insight to which combination of materials will prove optimal for a specified function.

## 2. Typical Protein and Polysaccharide Biopolymers

### 2.1. Protein Biopolymers

Protein biopolymer materials are produced from plants, animals and types of bacteria. These materials can arise from protein precursors that can be augmented by post-translational modification [30]. Protein precursors or pro-proteins can be located at the N or C terminus of the signal peptide [31]. They are important for protein folding. After the proteins are mature, properties, such as the structure and function, change drastically. Different types of proteins have been researched to act as biomaterials or combined with other proteins or polysaccharides to be applied to biomedical applications. This section will go more into detail about the following protein materials: Silk, keratin, soy, corn zein, wheat gluten, resilin, collagen, and gelatin.

#### 2.1.1. Silk

Silkworms, spiders, and some insects produce the protein silk, the toughest fiber found in nature [32,33,34]. Silkworm silks are primarily comprised of fibroin and sericin proteins while spider silks are composed of mainly glycine and alanine-enriched fiber proteins. Silk proteins are a naturally occurring biopolymer with favorable properties, such as mechanical strength, biodegradability, and minimal immunogenicity [32,33]. The mulberry silkworm *Bombyx Mori* spins large amounts of silk cocoons of consistent thickness while spiders produce tiny increments of silk of varying thickness to serve a particular function. These silk fibers demonstrate excellent mechanical properties like high tensile strength, flexibility, and resistance to compression [32]. The hydrophobic domain of silks allows for tightly packed beta sheet crystals to make up the structure. The larger hydrophobic domains interspaced with smaller hydrophilic domains are what gives silk its unique structural properties [34].

#### 2.1.2. Keratin

The protein keratin is an insoluble intermediate filament, which make up the bulk of adnexa of the epidermis, such as hair, nails, wool, and hooves. Keratin can be classified as “soft” or “hard”. Soft keratins are those that form loosely packed bundles of cytoplasmic intermediate filaments while hard keratins are embedded in a matrix of cysteine-rich proteins that structure epidermal appendages [35]. Both types of keratins have similar structures in that they consist of two chains, which each contain a central alpha-helical domain [36]. Keratin is a readily abundant protein source with biodegradability and biocompatibility capabilities. Due to their intrinsic ability to self-assemble and create porous and fibrous structures, they are often selected as a biomaterial for various applications [35]. Keratins are also able to possess cell-binding properties and serve as a site for cellular infiltration, attachment, and proliferation [35,37].

#### 2.1.3. Soy Proteins

A plant protein, such as soy, is mainly used for the storage of amino acids. The soy monomer’s amino residues are linked by amide bonds into polypeptide chains [38,39]. Soy proteins have been used previously as a synthetic replacement for plastics. While soy has excellent environmental properties, it lacks mechanical strength and water resistance properties [40]. There are three different types of soybean products used in polymer alternatives: Soybean whole fat (SF), soy protein concentrate (SPC), and soy protein isolate (SPI). Composites generally use SPI over the other soybean product due to it being a readily available resource from soybean, and it possesses properties, such as biodegradability and high strength, but it can be brittle and sensitive to water [41].

#### 2.1.4. Corn Zein

Zein accounts for about 80% of corn’s protein content [42]. Zein has had recent advances in serving as a biomaterial in the medical, pharmaceutical, and food industry fields [42,43]. It possesses important characteristics, such as biodegradability, biocompatibility, mechanical strength, and excellent fiber and film-forming capabilities. Zein is amphiphilic, so it possesses hydrophobic and hydrophilic properties. Zein can be divided into three classes based on solubility and molecular weight: Alpha-, beta-, and gamma-zein [42].

#### 2.1.5. Wheat Gluten

Wheat gluten protein has been posed as another polymer alternative. Wheat gluten is a byproduct of wheat starch and is a very abundant biodegradable and renewable agricultural resource. Gluten is a mixture of monomeric and polymeric materials, gliadin and glutenin, respectively [44]. Gliadin impacts the viscosity of the gluten while glutenin amounts can change the elasticity [44]. Other favorable properties of gluten include stability against water and heat, biodegradability, and the ability to form fibers easily. One limitation of wheat gluten is that it does not exhibit a high mechanical strength [45].

#### 2.1.6. Resilin

Resilin is found in the cuticles of insects, and is a rubbery protein that exhibits high elasticity due to solely containing amino acid residues. It also has more hydrophilic qualities due to the basic and acidic nature of the amino acid residues. Its rubbery property makes it susceptible to high mechanical strength. It is able to undergo high deformation and return to its original state under stress [46]. Resilin is considered an elastomeric protein because of its extensibility and elasticity. Its hydrophilic biopolymer network give value to this type of protein, especially in applications where high mechanical strength is needed [47].

#### 2.1.7. Collagen and Gelatin

Collagen serves as the most abundant protein in vertebrates and invertebrates [48]. Currently, there have been 27 different types of collagen identified [48]. Collagen is the main fibrous protein component in bones, cartilage, and skin. From collagen, the protein gelatin can be produced. This fabrication is from the partial hydrolysis of collagen or by producing a heterogenous mixture of polypeptides from collagen, which are produced by destroying cross-linkages [48,49]. A single collagen molecule contains three alpha chains with over 1000 amino acids. A property of collagen is that it is subject to post-translational modifications [50]. Collagen is insoluble while gelatin is very strong and has thermal stability [49]. The combinations of these materials have been useful in medical applications, such as drug delivery and implants [51,52].

### 2.2. Polysaccharide Biopolymers

Polysaccharide biopolymer materials are those found abundantly in nature and have been recently exploited for their excellent structural properties to form various composites. Like proteins, polysaccharides also have precursors [53]. Genes can form the precursor polysaccharides and will also be influenced by spatial and development changes in the nearby cells [53,54]. After modification, precursor polysaccharides will activate and possess the defined properties of their subsequent polysaccharide [55]. Because of their strong structures, they have been proven to excel as biomaterials. The following polysaccharides are explored deeper in this section: Cellulose, chitin and chitosan, starch, pectin, alginates, and hyaluronic acid.

#### 2.2.1. Cellulose

Cellulose is a type of polysaccharide found abundantly in nature and is easily chemically modified, which provides many advantages [56]. Cellulose forms the structural basis in plants, which makes it the most abundant renewable resource on the planet [57]. As a biomaterial, cellulose has served as wound dressings and in the form of hydrogels for orthopedic applications [58]. Favorable properties include high tensile strength and biocompatibility. Different means of enhancing its properties has been explored, such as phosphorylation or bacterial synthetization, which increase its bioactivity [57,59].

#### 2.2.2. Chitin and Chitosan

Chitin serves as a major structural component of invertebrates, insects, and fungi [60]. It is an extremely abundant biopolymer, right after cellulose. In its purest form, it is insoluble in water. Its structure is a highly linear and it is a highly crystalline polymer [60]. The material chitosan can be found in a few fungi species, and is mainly produced through chitin deacetylation. Due to its high degree of crystallinity, the materials are extremely stable through hydrogen bonding [61]. These materials contain no antigenic properties, which makes them biocompatible as well as eco-friendly [62,63,64].

#### 2.2.3. Starch

Starch is an abundant polysaccharide that is found in the roots, seeds, and stems of various plants and crops [65]. Starch is constructed of anhydroglucose units and subsequently comprises two different polymers: Amylose and amylopectin. While starch presents a few disadvantages, such as low mechanical strength and high hydrophilicity, it has demonstrated good biodegradability and cell seeding capabilities [66]. Therefore, starch has excellent structural capabilities for biodegradability and biocompatibility. Starch is relatively easy to modify, which makes it suitable for chemical enhancements to improve upon its weaker qualities [67].

#### 2.2.4. Pectin

Pectin is a carbohydrate material derived from plant walls, mainly as a citrus byproduct [68]. Pectin has excellent gelation properties. It is also hydrophilic in nature with many functional capabilities [69]. It can be divided into three main regions: Smooth, hairy, and branched. The gelling property as well as solubility is dependent upon the esterification of galacturonic acid residues. Because of its gel-forming abilities, it has been recommended for the use of delivery bioactive agents. Pectin is non-toxic, and high in fiber content, which has made it successful in the food industry [70].

#### 2.2.5. Alginates

Alginates are an important polysaccharide and can be found in algae species and soil bacteria [71]. Being one of the most biosynthesized materials, alginates are naturally hydrophilic and anionic [72]. Alginates have an excellent ability to store and retain water, as well as stabilizing and gelation properties. They are biocompatible and immunogenic, which makes them applicable to biomedical applications [73]. Chelation properties also make alginates favorable in drug delivery systems or tissue regeneration [72].

#### 2.2.6. Hyaluronic Acid

Hyaluronic acid is a natural linear polysaccharide found in the extracellular matrix of animals. This material is naturally biocompatible, biodegradable, and lacks immunogenicity [74]. Its structural properties give it the ability to mediate cell signaling, provide wound repair, and declare matrix organization [75].

## 3. Fabrication Methods

The intent of use of the designed protein–polysaccharide composite material can guide which fabrication technique should be utilized. For example, if a biocomposite was to be created to deliver an anti-cancer component to the colon, it would be necessary to develop the components with a specific shape and structure so that they are not susceptible to disruption or digestion within the mouth, stomach, and small intestine, but will break down in the colon [76]. Various fabrication techniques for protein–polysaccharide composites are coacervation, phase separation, electrospinning, and cryogenic treatment. Each technique has its own set of advantages and disadvantages, which can be viewed in Table 1. 

### 3.1. Coacervation

**Figure 2:**
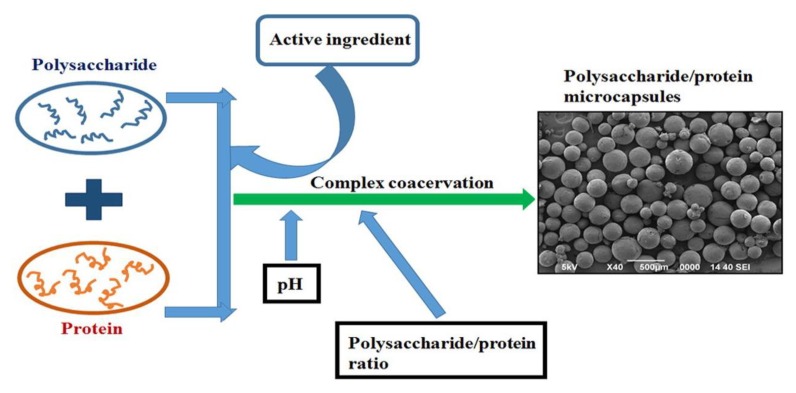
Complex coacervation process showing the combination of protein and polysaccharide (reproduced with permission from [84,85]. Copyright Elsevier, 2010 and 2017).

Spontaneous formation of biopolymer-based particles is achieved most notably through coacervation [76]. Coacervation is a chemical process shown in Figure 2, where biopolymers of opposite charge interact on a very short scale, thus producing associative complexes [77]. By utilizing pH values and biopolymer conditions, a soluble complex can be formed from just a few interacting molecules [77,78]. Recent studies have shown interactions of soluble complexes between globular proteins and polysaccharides near or higher than the isoelectric point [79]. These soluble complexes are held together mostly through their isoelectric interactions at specified and localized charged segments [80]. Therefore, by changing the solution conditions, one can easily reverse the formation of these complexes. If the desired structure needs to be retained, certain methods are required to compact the complex components to increase their isoelectric bonds within the agglomerate [76]. A proven method of such cross linking has been seen through the use of aldehydes, such as glutaraldehyde [81]. Biopolymer particles can be characterized by their physical properties and their stability in varying environmental conditions. These physical properties, such as size or charge, can be observed through experimental techniques, such as light scattering [82] and electrophoretic mobility [83]. The stability of the protein–polysaccharide biocomposite is dependent upon various conditions, such as pH, ionic strength, heating, and freezing. The stability relies on the compound’s composition, surface characteristics, and its structural integrity.

### 3.2. Electrostatic Spinning

Electrospinning is a fabrication approach for numerous composite polysaccharide–protein solutions through the use of electrostatic forces to create thin fibers from the biopolymer solution. Melting can also be used during the spinning, which allows the fibers produced to have a thinner diameter (nanometer to micrometer) along with an increased surface area compared to those obtained from conventional spinning processes. In order for electrospinning to occur, a DC voltage of about 15–50 kV is necessary to generate the spinning. This method is based on the principle that mutually strong electrical repulsive forces will overcome the weaker forces correlated with surface tension in the charged biopolymer solution. There are multiple other techniques similar to electrospinning, such as pesticide sprayers and electrostatic precipitators [59]. Currently, there are two standard electrospinning techniques, vertical and horizontal.

**Figure 3:**
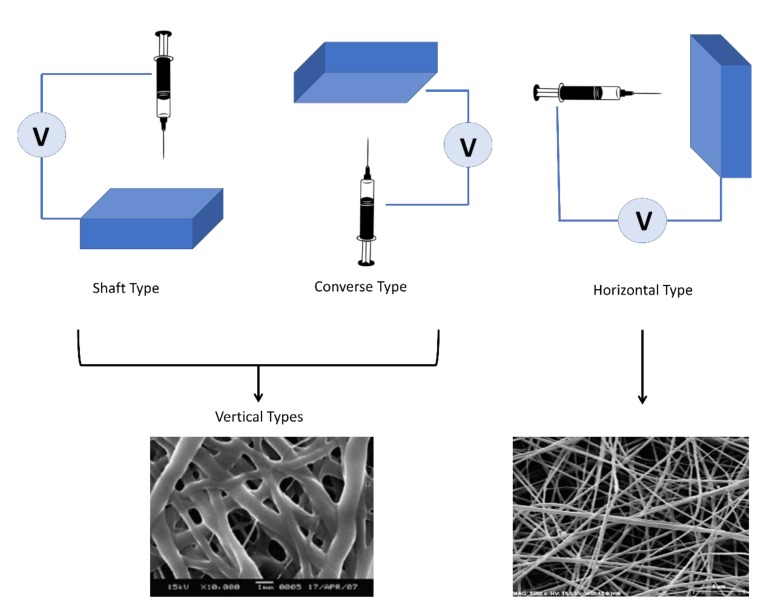
Schematic of electrospinning systems with respective orientation and SEM images of the fibers for the different protein–polysaccharide systems (left: collagen–chitosan composites; right: amaranth protein–pullulan blends) (reproduced with permission from [86,87], Copyright, Elsevier 2010 and 2013).

Horizontal and vertical electrospinning were named according to their geometric layout, which influences the physical outcome of nanofibers. Vertical electrospinning has two types known as shaft type and converse type [86,87]. Shaft-type electrospinning has been shown to yield the thinnest fibers due to the effect of gravity strengthening the effects of the electric field, causing the fibers to extend much more sufficiently. However, it also has the widest size distribution of the fiber diameter, making this method less controllable. The converse type outputs the thickest fibers but has the smallest diameter distribution, making for a controllable but large fiber production. The horizontal-type system shows fibers that are in between what is generated from the shaft type and the converse type [88]. Figure 3 represents the orientation of each electrospinning technique with images of the fibers from different protein–polysaccharide systems.

### 3.3. Film Formation and Phase Separation

Binary biopolymer solution experiences various events and stages. The solution can stay in a homogenous phase. It can also separate in two phases, where the two polymers remain away from each other. Lastly, the biopolymers can associate and consequently precipitate or gelatec [89]. This system can be represented in a phase diagram. The binodal will separate a compatible region (one phase) from an incompatible region (two phase). This incompatible region can be divided into a metastable region, where the mixture separates through growth and nucleation, and an unstable region, where the mixture then separates through spinodal decomposition [90,91]. Spinodal decomposition is characterized through spontaneous formation of single-phase domains, which will grow and become purer with time in order to achieve an equilibrium-like state. Phase separation through nucleation and growth can be understood as formed droplets that are irregularly spaced, appear at different times, have an array of size distributions, are at equilibrium, and will continuously grow with time [92,93,94]. In order for separation to be initiated through nucleation, an activation energy is required, unlike spinodal decomposition, which occurs spontaneously [92].

A study by the Swedish Institute for Food and Biotechnology showcased the prowess of the custom films and how they were created by combining the gas barrier of the hydrophilic high amylose maize starch with a hydrophobic protein zein [90]. Since two polymers may not be miscible, the phase separation of this mixture is crucial for the ultimate film structure and properties. They induced phase separation through cooling, which they observed as growing droplets of the starch with small aggregates, and through solvent evaporation. This was studied with real-time confocal laser scanning microscope. Their study showed that solvent evaporation had a greater effect on phase separation and the early stages caused the starch to form large aggregates. Smaller droplets were also intertwined with other droplets or large starch aggregates [90].

### 3.4. Hydrogel and Gel Formation

Polysaccharides or proteins can form gels due to changes in intermolecular bonding—this gelation is induced and is sensitive to temperature [95]. The thermo-reversibility is the most notable characteristic of gelatin [96]. Some examples, such as agarose, amylopectin, and partially hydrolyzed collagen, also possess this key trait [96]. The formation of the helices in polysaccharides or proteins drives the growth of helical aggregates [95].

A second class of temperature-sensitive materials incorporates the use of a lower critical solution temperature system. A homogenous solution can be obtained at low temperatures, and upon heating, hydrophobic groups swarm together, which induces phase separation and hydrogel formation. This gelation is propagated by an increase in entropy when the hydrogel forms, and the entropy increase results from a large amount of water molecules that are released by the hydrophobic part of the polymer [97]. Gelation occurs spontaneously with the addition of heat because the entropy overcomes the enthalpy that is unwanted by the biopolymers [95].

**Figure 4:**
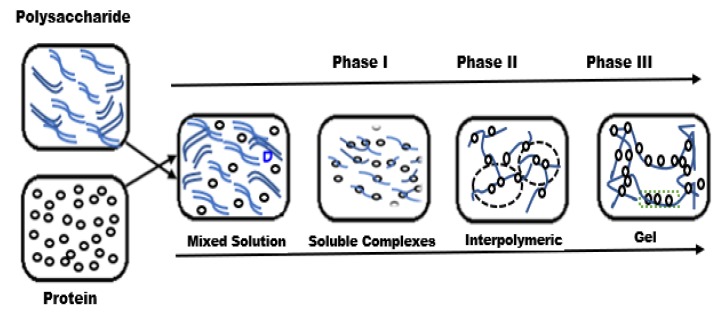
Formation of protein–polysaccharide gel complexes at different stages (reproduced with permission from [102] Copyright Elsevier, 2017).

Hydrogels can be synthesized at ambient and cryogenic temperatures [98]. Cryogenic treatments fabricate highly porous hydrogels that are better known as “cryogels” [98]. In gel-forming systems, cryogenic temperatures can also be used to enact cryogelation [99]. This process requires the crystallization of the bulk of low molecular weight liquid that is involved in the gel formation. Due to this crystallization, the total volume of the non-frozen liquid microphase is less than the initial reaction volume [98]. The concentration of the polymer or monomer in the non-frozen liquid microphase is far greater than the initial concentration. The biocomposite gel phase can be formed during any of the previous steps in the cryogenic treatment process: During initial freezing, during storage of the frozen samples, or during the thawing of frozen materials [97,100,101]. Figure 4 provides a typical formation process of protein–polysaccharide gel complexes at different stages [102].

## 4. Impact of the Solvent and Post-Treatment on the Material Structure and Property

### 4.1. Solvents, Miscibility, and Interactions

The improvement of cell adhesion can be attributed to the presence of proteins in blends due to the increase in binding sites [103]. The native physical structures of proteins have limited possible intra- and interchain interactions due to their linear structures [104,105]. Polysaccharides play the role of the structural component in these complexes. There are a variety of different forces that can be found in molecular protein–polysaccharide interactions, such as electrostatic, hydrophobic, hydrogen bonds, steric interactions, and Van der Waals [106]. Different parameters, such as temperature, can influence the hydrogen and hydrophobic bindings, whereas the pH and ionic strength can also induce protein denaturing [103].

**Figure 5:**
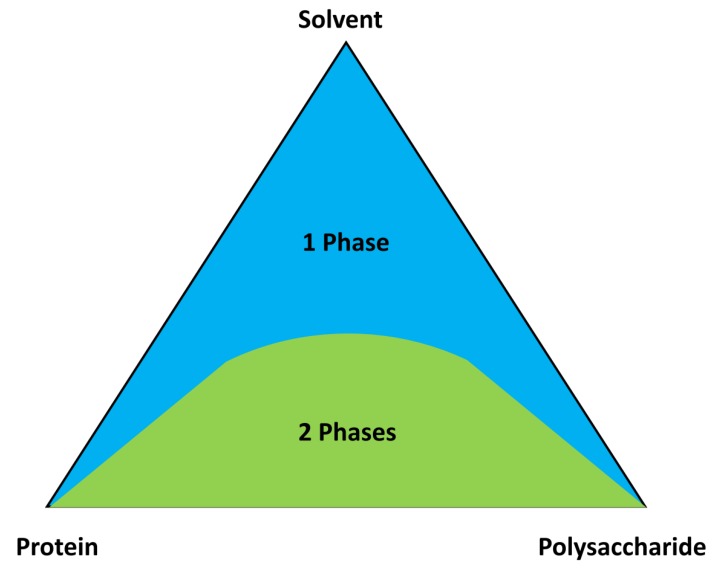
Ternary-phase diagram of a protein/polysaccharide solvent system.

As an example, salt ions can be introduced to a solvent to induce electrostatic interactions between protein chains and polysaccharide to obtain blends of varying miscibility. The mixing ratio points of protein:polysaccharide:solvent can be obtained by mixing various volumes of a component and then plotted onto a phase diagram, as shown in Figure 5. The miscible solutions are identifiable in the single-phase region whereas the phase-separated solutions are respectively in the two-phase region. Quantitative analysis of the miscibility can be conducted by calculating a miscibility parameter and reduced viscosities via methods developed by Krigbaum and Wall [105] and Garcia et al. [104]. Due to the high amounts of solvent and salt the solutions had, an almost electroneutral characteristic was apparent, which corresponds to the net change of all blends nearing zero in the single-phase region.

Since proteins are surface active, they also have a large role in the creation and preservation of emulsions when interacting with polysaccharides, either through hydrophobic or electrostatic interactions. Whereas polysaccharides are naturally hydrophilic, they generally remain in an aqueous phase, which helps in tuning the rheology. Good miscibility is indicated by the positive miscibility parameter for all blends. This miscibility is accounted for due to the electrostatic interactions between calcium ions and chains, thus leading to a more stable mixture.

#### 4.1.1. Ionic Liquids as Solvents

Solvents have a significant impact on the dissolution of materials, specifically for the creation of protein–polysaccharide composites, therefore requiring unique solvents. The dissolution process is essential as it provides a framework to combine and blend the materials making up the composite [22]. Solvents, such as ionic liquids, have shown excellent capabilities in creating such composites. These liquids are compounds with melting points below 100 °C and composed completely of ions [106]. Ionic liquids are extremely universal, and the properties of the constitutive cations can undergo unlimited structure transformations. The possibilities for cations and anions to interact are virtually unlimited in creating an ionic liquid. Because of this, the properties of the liquids yield excellent and adjustable chemical properties, such as polarability, miscibility, and solubility [107]. Ionic liquids as solvents have properties, such as stability, reusability, and conductivity [23]. Ionic liquids are generally preheated in an oven to remove any excess water before being used as a solvent [22].

The structure of an ionic liquid is a substantial asymmetric cation and a weakly coordinated anion. As previously stated above, this structure allows for a wide range of possibilities in forming composites [23]. DeFrates et al. and Stanton et al. describe the dissolution process for creating a cellulose–silk composite [22,23,108]. The dissolution process occurs by the ionic liquid’s anion attaching to the hydroxyl groups in order to form hydrogen bonds, which disrupts the natural hydrogen network. The cation then associates with the polysaccharide’s oxygen atoms and CH group [22,23]. To do this, a solution of the cellulose and silk with the ionic liquid was created, with 10% of the composite materials and 90% liquid. The liquid is stirred on a hot plate at 100 °C and placed in a silicone oil bath to ensure a properly distributed temperature [22]. After dissolution, the cellulose becomes disordered and extends its fibrils for silk molecules to attach to and create the composite. It will retain its cellulose crystallinity property but have interspersed silk molecules [108]. After dissolution is complete, the composite is coagulated with water or methanol to form a biofilm [22].

#### 4.1.2. Organic Solvents

Protein–polysaccharide composites are traditionally treated in organic solvents, which are known as a type of volatile organic compound (VOC) [109]. VOCs evaporate at room temperature and include organic solvents, such as alcohols, esters, ketones, acids, ethers, aromatic compounds, and hydrocarbons [109]. Isopropyl alcohol can regenerate the protein–polysaccharide complex by coating the water-soluble polysaccharide with the water-insoluble protein, preserving the properties of both the protein and polysaccharide blend [110]. In one study, trifluoroacetic acid (TFA) and dichloromethane (DCM) organic solvents were used to dissolve protein–polysaccharide composites of silk fibroin and chitosan for electrospinning [111]. In this method, the organic solvents were evaporated from the composite successfully and were not detected using the infrared spectroscopy for analysis of the resulting nanofibers. The versatility of organic solvents was shown in this study, where a mixture of ethanol and ammonia was used to chemically treat the protein–polysaccharide composite to remove the residual TFA and alter its conformation [111].

In general, organic solvents can often produce harmful side products, which do not make them favorable to the environment [23]. If used in a certain manner, organic solvents can significantly alter the molecular weight of delicate materials, such as silk proteins, which can destroy their favorable properties [23].

#### 4.1.3. Other Solvents

Polysaccharides can only be dissolved in certain solvents because of their structures [111]. The amorphous and crystalline regions connected by hydrogen bonds require polar solvents to be broken down [111]. Polar solvents are extremely flammable, toxic, or possibly corrosive, which makes them particularly unattractive [111]. Some materials, such as soy, are soluble in water. Cao et al. dissolved soluble soy protein isolate in deionized water with the addition of glycerin [112]. From this process, a soy–gelatin composite was created [112]. Heat can denature proteins and is also used during water dissolution to produce a stronger complex [113].

### 4.2. Post Treatments

Post treatments on protein–polysaccharide complexes have been done chemically or physically to improve the material properties of the composite [114]. Depending on the desired application of the composite, the method can be tuned to reach the desired outcomes. Alternate drying or freezing conditions can be used with different timings to produce two completely different physical characteristics. Techniques such as these are commonly used in industry. In one study, a protein–polysaccharide composite of collagen and sodium alginate with a sponge-like appearance had water removed by freeze drying to control the pore size in the samples [115]. The small porous composites of about 20 μm were shown to be useful as a wound contact layer to allow the penetration of fibroblasts into the matrix, whereas the production of composites with larger pores of around 100 μm is desirable for materials used as a component of a burn dressing. Chemical dehydrothermal crosslinking and treatment with crosslinking agents, such as aldehydes, also increased the stability of the collagen–alginate composite by 50% after seven hours of soaking compared to the untreated complexes [115]. Physical and chemical post treatments are also done on different protein–polysaccharide composites.

#### 4.2.1. Chemical Coagulation

Chemical treatments of protein–polysaccharide composites can induce permanent structural changes to these composites. Some examples of chemical treatments include treatment with water, methanol, and other solvents [116].

Water treatments are primarily used to rinse and dissolve organic materials from the composites [116]. Two types of interactions can occur with water treatments: Segregative or associative phase separation. The segregative phase is when the proteins and polysaccharides that have been combined have synonymous changes. With the associative phase separation, different types of constructs can be formed and used for hydrogels and coacervates [102]. On the other hand, methanol treatments provide a way to chemically or physically crosslink composites. One method of this is forming films, drying and immersing them in a methanol solution overnight [102]. This allows for the film to become crosslinked with other films. Methanol works primarily as a dehydrating agent. These films are also washed several times with pure methanol at room temperature [5]. Liu et al. performed this cross-linking method with a pectin–gelatin composite. The cross-linking showed a very oriented, heterogeneous structure [5]. The protein portion of this structure appeared to dominate the material, with pectin dispersed throughout [5]. Lastly, the addition of another solvent can initiate a reaction. In a study performed by Chen et al., a gelatin–chitosan mixture was mixed with tyrosinase to oxidize the composite material [117].

#### 4.2.2. Physical Treatment and Properties

Physical treatments are also used to change the structure in protein–polysaccharide composites. These changes include heat treatments, mechanical stretching, and mechanical compressing. The use of mechanical treatments can enhance the mechanical properties of the composite.

Heat has the ability to denature proteins and complex formation between the electrostatic interaction of the polysaccharide and protein. These interactions have the ability to form hydrophobic or disulfide bonds [114]. Another reason heat is used is the composites have the ability to produce biocrude solid products. These products can be used for biofuel applications, which can present alternatives to traditional methods. The extent of the temperature increase affects the amount of solids produced and is dependent upon the proteins and polysaccharides used [116]. Tensile or compression tests are used to determine the strength of a construct. In one particular case of a pectin–soy composite, Mariniello et al. used a transglutaminase treatment on the composite to enhance its mechanical properties [118]. In this study, the strength was improved by close to 82%. The tensile strength of the composite was roughly 6.8 MPa and after the addition of transglutaminase, the tensile strength increased to 12.4 MPa [118]. Another form of mechanical manipulation is mechanical breakdown, which is used to produce small particle gels. Leon et al. described how a study used a food processor to mechanically reduce the size of nanoparticles for a wheat–alginate composite [119].

## 5. Novel Applications of Protein–Polysaccharide Materials

Natural polymers are ideal biomaterials due to their biocompatibility to their host environment, tunable mechanical properties, and their ability to mimic the biochemical aspects found within the body. Protein–polysaccharide composite materials have provided many possibilities to enhance biological functions and improve current technology. When used as a biomaterial, they could potentially improve the fabrication of artificial tissues or implants for tissue regeneration, drug delivery, and other applications.

### 5.1. Tissue Regeneration

Tissue regeneration has been a viable approach to restoring biological functions of damaged tissues and organs. In an ideal model, a combination of cells, biomaterials, and chemical and physical factors must be constructed to mimic the targeted tissue.

Protein–polysaccharide composite materials have been used to promote the growth of healthy tissue under proper conditions. Controlling the composition and biochemical properties of fabricated tissues is a challenge due to the highly complex structures of native tissue. However, within tissue regeneration, it is common to address the components that contribute to tissue complexity. Some applications include engineering the extracellular matrix (ECM), wound healing applications, cardiac tissue regeneration, bone regeneration, or liver tissue engineering. Ding et al. fabricated natural scaffolds to simulate the ECM using silk fibroin and chitosan for future applications in tissue regeneration [120]. The nanostructures demonstrated an improvement of biocompatibility, cell proliferation, and neovascularization. Rosellini et al. mimicked the chemical composition and molecular interactions found in the native cardiac ECM with scaffolds made of alginate and collagen/gelatin material [121]. The alginate and gelatin scaffolds were superior biochemically and mechanically when compared to alginate and collagen scaffolds. Wang et al. addressed a common application of wound healing with a chitosan–alginate polyelectrolyte (PEC) membrane [122]. In vivo testing with rat models resulted in maturity of the epidermal structure and a reduction in inflammation in the dermis after a few weeks of treating an incisional wound. Li et al. fabricated a multicomponent composite consisting of nano-hydroxyapatite/chitosan–gelatin for bone tissue engineering [123]. The composite demonstrated promising mechanical, physico-chemical, and biological properties for bone regeneration. Tahmasbi Rad et al. developed a scaffold construct for live tissue engineering that included chitosan, gelatin, hyaluronic acid, collagen, and poly(3,4-ethylenedioxythiophene) (PEDOT) [124].

Engineered extracellular matrix scaffolds have gained recognition due to their physical property control, chemical composition, and mechanical properties. Biomimetic materials, such as natural protein and polysaccharide composites, have been used to mimic the proteins, glycoproteins, and glycosaminoglycans found in the ECM. Natural materials do present limitations regarding the mechanical integrity, risk of pathogen transfer, and uncontrolled degradation rates [89]. The engineered ECMs are commonly represented through the fabrication of scaffolds, which are three-dimensional, porous, and tunable biomaterials used to remodel or restore the functions of damaged tissues. An important necessity for engineered scaffolds is to have high porosity and interconnectivity of these pores. A structurally sound scaffold will increase cell seeding and growth. Another goal is to preserve tissue volume and facilitate the circulation of nutrients and waste products [121]. In addition, the hydrophilicity of scaffolds is important for the absorption of bodily fluid and transfer of cell nutrients and metabolites [123]. Through in vitro studies, scaffolds are typically seeded with cells found at the implantation site to observe cell integration. The ability to control the composition and biochemical properties of a scaffold allows for improvement of the cell performance.

**Figure 6:**
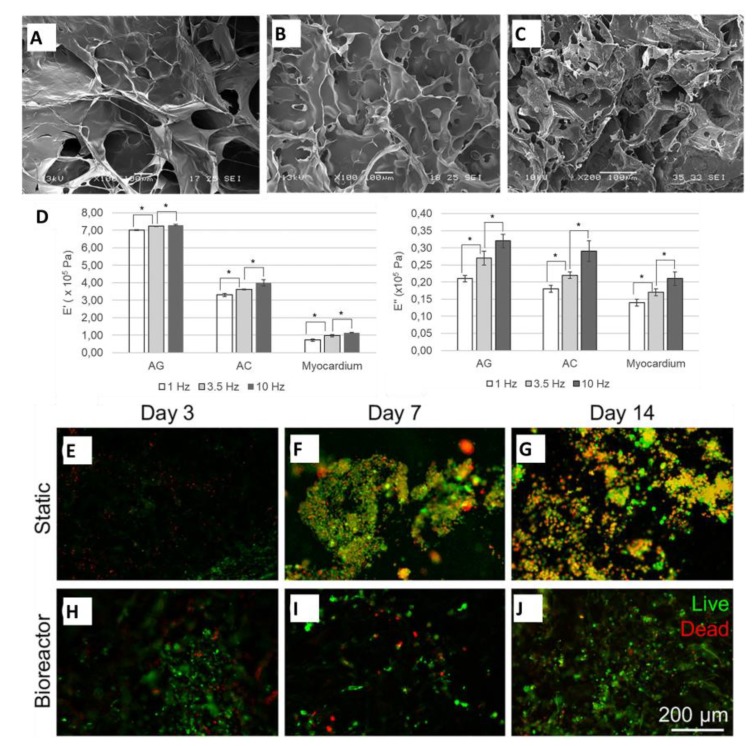
(**A**–**C**) SEM images (scale: 100 μm) of the different protein–polysaccharide materials: (**A**) Alginate/gelatin (**B**) Alginate/collagen (**C**) Decellularized porcine myocardium. (**D**) Mechanical characterization of these protein–polysaccharide materials showing different storage and loss modulus. (**E**–**J**) Fluorescence micrographs displaying cardiomyocytes response to alginate/gelatin scaffolds at different days under static and dynamic conditions (**A**–**J** reproduced with permission from [121], Copyright Wiley, 2017).

Rosellini et al. developed a cardiac ECM substitute scaffold using two different protein–polysaccharide combinations, either alginate with gelatin or alginate with collagen (Figure 6) [121]. Based on the mechanical properties, the development of both scaffolds indicated a potential for improved cardiac tissue engineering by reducing heart wall stress. Initially, the alginate solution was physically crosslinked with chitosan or gelatin solution by mixing and stirring at room temperature. Both mixtures were set at the same weight ratio: 20:80 polysaccharide to protein. After the composite was freeze-dried to form sponge-like structures, a crosslink between GTA vapor and the protein components was later treated with calcium ions to crosslink the alginate component. To avoid cytotoxicity, the scaffolds were thoroughly rinsed to eliminate any toxic additives needed for the fabrications. Comparing the three materials, decellularized natural porcine myocardial tissue, alginate–gelatin, and alginate–collagen mixtures, the morphological, physicochemical, functional, mechanical, and biological properties were characterized by multiple assessments [121].

Based on the superior behavior of alginate–gelatin, it was concluded to be more suitable for cardiac tissue engineering applications. The morphology of the materials was analyzed with a scanning electron microscope (SEM). The alginate–gelatin and alginate–collagen demonstrated an ideal highly porous, homogenous structure compared to the decellularized myocardium. Numerically, the scaffolds ranged from 49–60% porosity while the myocardium was at 13% porosity [121]. This is a strong indication for proper cellular behaviors within the scaffolds because it deviates significantly from the percentage of porosity of the native tissue. Nevertheless, the scaffolds and decellularized myocardium have elastic behaviors based on the significant difference between the loss and storage modulus. The interactions of the alginate and gelatin produced a higher loss and storage modulus when compared to the other materials. The alginate–gelatin began to show the predominant properties within the in vitro degradation testing, swelling test, and cellular response. The alginate–gelatin scaffold was later investigated for its cellular response under dynamic conditions because during static conditions, the alginate–gelatin scaffold was a better support for the C2C12 myoblasts to adhere to, grow, and differentiate [121]. In a microfluidic bioreactor, the alginate–gelatin scaffold was able to positively interact with cardiomyocytes under dynamic environments. As a result, a high viability of cardiomyocytes was observed.

**Figure 7:**
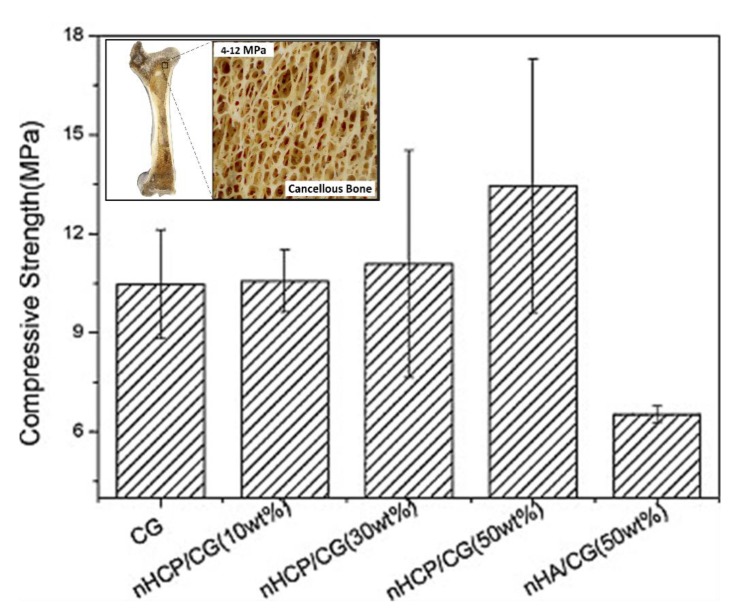
Compressive strength of the different protein–polysaccharide composites: Bulk composite of nHCP/CG (nHA/chitosan–pectin/chitosan–gelatin) is similar to that of cancellous bone (reproduced with permission from [123], copyright Elsevier, 2011).

An ideal composite material for bone regeneration should be biodegradable to allow the replacement of new bone to grow properly, mechanically robust to temporarily support the new bone formation, and biocompatible to facilitate early mineralization. Li et al. fabricated bulk multicomponent polysaccharide/nano-hydroxyapatite (nHA) composites to investigate the physico-chemical, mechanical, and biological properties suitable for bone regeneration (Figure 7) [123]. The multicomponent composite consisted of a combination between nHA and chitosan, gelatin, and pectin. There were other combinations used for comparison, such as nHA/chitosan–gelatin and nHA/chitosan–pectin. Similar to the other materials, the bulk composite was produced using nHA/chitosan–pectin mixed with chitosan–gelatin through mineralization. It required a more extensive procedure, using glutaraldehyde as the crosslinking agent [123]. All the composites were fabricated to form a scaffold through a freeze-dry method.

For bone regeneration, the porosity plays a key role by controlling the mass transfer of nutrients and metabolic wastes to cells. It was observed that the nHA/chitosan–gelatin scaffold had less porosity than the bulk composite of nHA/chitosan–pectin and chitosan–gelatin. However, when the weight percentage of the bulk composite varied, it was shown that the greater ratio of nHA/chitosan–pectin to chitosan–gelatin decreased the porosity [123]. The lower concentration of nHA/chitosan–pectin in the bulk composite led to beneficial cell migration and vascularization [123]. These properties are also important components for new bone formation. For the 50 wt.% bulk composites, pre-osteoblasts were observed to proliferate and migrate more efficiently on the outer surface, inner, and corner of pores than the other composites [123]. The multilayered pre-osteoblasts were able to grow in a circular manner throughout the pores while supported by a steady chemical composition from the bulk composite. The mechanical composition of the bulk composite is described to be similar to cancellous bone based on the compressive strength. Cancellous bone has compressive strengths between 4 and 12 MPa, and the bulk composite was found to be approximately 13.45 MPa [123]. As a result, the multicomponent composite of nHA/chitosan–pectin mixed with chitosan–gelatin demonstrated promising mechanical stability, biocompatibility, and great biological response to pre-osteoblasts, indicating a desirable material that could be tuned for the requirements needed for bone regeneration [123].

Liver regeneration is challenging to replicate due to its highly sensitive properties. After isolation of a primary hepatocyte, the phenotype can quickly change based on the surrounding environment. In addition, hepatocytes need to be anchored and be very interactive with the ECM for maintenance of their normal functionality [124]. Aside from cancer and stem cells, liver cells have the greatest depolarization, insinuating a need for a membrane voltage component in a composite. Tahmasbi Rad et al. fabricated a suitable liver regenerative scaffold to mimic the ECM components and interactions between the scaffold and cell surface receptors [124]. The biomaterial composition plays an important role in replicating liver-specific functions and mechanical stability. Chitosan, gelatin, type I collagen, hyaluronic acid, and a conducting polymer named poly(3,4-ethylenedioxythiophene) (PEDOT) were combined to create a variety of scaffolds. Each material adds an advantage to the composite, especially the PEDOT, which improves the electrical signaling between the hepatocyte and the scaffold. The other materials also improve the chemical and biological signaling by providing an appropriate environment for the hepatocytes to grow in.

A homogenized mixture of five varied samples of each material was fabricated through freeze-drying and crosslinking agents [124]. Each mixture was allocated in a pre-designed mold to acquire a specific size. The FTIR was utilized to ensure the bond connectivity and functional groups in the scaffold. SEM images identified which mixtures were superior for liver tissue scaffolds. The addition of PEDOT was observed to reduce the porosity size of the scaffold from 350 µm in gelatin/chitosan to about 200 µm in gelatin/chitosan/PEDOT [124]. Hyaluronic acid contributed to the porosity of the scaffolds to have order and formation between parallel walls. However, without the addition of hyaluronic acid, the pores were more amorphous and circular like [124]. In addition to porosity, the hydrophilicity of the scaffold was affected by collagen or PEDOT. The strong bonding between gelatin, chitosan, and collagen was considered as the reasoning to the reduction of swellability in the scaffold [124]. The FTIR supported the explanation by demonstrating greater peaks in the collagen-based mixtures, which indicated more bonds. This observation correlated with the degradation rates of the scaffolds. The gelatin, chitosan, and collagen mixture had a lower degradation rate than gelatin, chitosan, and hyaluronic acid. Therefore, it was concluded that collagen decreases the biodegradability rate by lysozyme [124]. A lysozyme is an enzyme that catalyzes the destruction of cell walls.

The scaffolds were demonstrated to be supportive of adherence and proliferation of the GS5 liver cells. The analysis from the SEM images resulted in an even distribution of cells throughout the pores of the scaffold, indicating good cellular migration, infiltration, and attachment [124]. This suggests there is potential cytocompatibility of the scaffolds. The addition of hyaluronic acid to the scaffolds seemed to provide anchorage sites for the liver cells. Overall, like many other applications, liver regeneration scaffolds are influenced by the morphology, mechanical, electrical, chemical, and biological properties to optimize an appropriate biomaterial composite.

### 5.2. Drug Delivery and Nanomedicine

Drug delivery systems are devices or pharmaceutical compounds that achieve delivery and release of therapeutic agents in the body through targeted and controlled strategies [125,126,127]. The routes of drug delivery are becoming more prevalent due to the adverse events caused by drug interactions with parts of the body that are not included in the targeted delivery [125]. The delivery systems vary from liposomes, nanoparticles, microspheres, and gels. An ideal system will have a material composite with mechanical stability, biocompatibility, and degradation within an acceptable period [125]. The general goal of drug delivery is to achieve successful transfer and release of drugs in a predetermined manner [128].

Raj et al. investigated the possibility of utilizing cisplatin-loaded cassava starch acetate–polyethylene glycol (PEG)–gelatin nanocomposite for anticancer drug delivery [129]. The nanocomposite drug release system was based on pH and time. Results from the in vitro studies showed there is a potential for cisplatin-coated nanocomposites for future in vivo studies. Tran et al. developed a method to improve the mechanical properties and versatility of composites made of keratin, cellulose, and/or chitosan [130]. The composite materials were synthesized through an environmentally friendly process and could be applicable for treating the ulcers of diabetic patients [130]. Chang et al. fabricated a chitosan–pectin composite for the release profiles of proteins for probable oral controlled release carrier applications [131]. Elia et al. produced a novel hyaluronic acid–silk hydrogel as a drug delivery vehicle [132]. The new hydrogel is capable of storing drugs, cytokines, growth factors, cells, and other therapeutic agents [132]. Cong et al. crosslinked alginate hydrogel with chitosan micelle to create a pH-sensitive drug delivery system [133].

**Figure 8:**
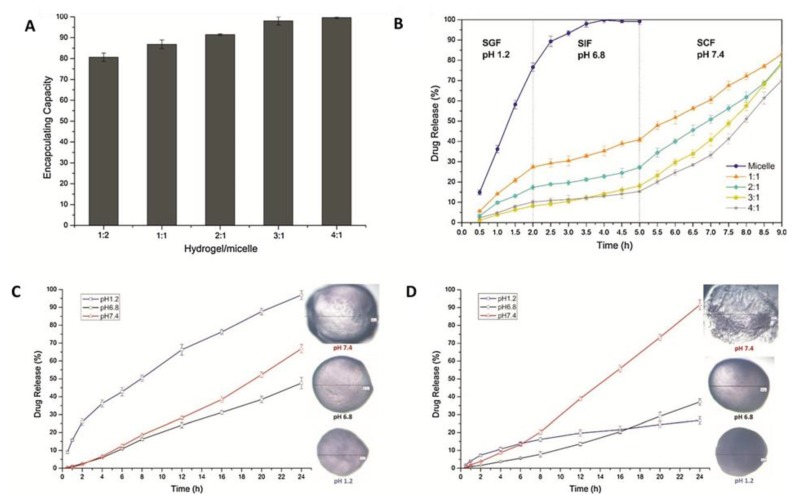
(**A**) The encapsulating capacity of different hydrogel/micelle systems. (**B**) In vitro drug release profiles of micelles and different hydrogel/micelle systems. (**C**) The drug release profiles of hydrogel/micelle = 1:1 and (**D**) hydrogel/micelle = 3:1 systems in simulated gastric fluid (SGF), simulated small intestinal fluid (SIF), and simulated colonic fluid (SCF), individually (**A**–**D** reproduced with permission from [133], copyright Elsevier, 2018).

Some intravenous injection applications for drug delivery have utilized polymeric micelles (Figure 8) [133]. Polymeric micelles have hydrophobic cores with hydrophilic shells for storage and protection for the internal substrate from the surrounding environment [133]. Cong et al. fabricated a pH-sensitive and drug-loaded micelle, using emodin encapsulated by chitosan within a sodium alginate hydrogel. Crosslinked micelles through chemical reactions or UV irradiation are more mechanically robust and effective for targeted release when compared to polymeric micelles [133]. The empty chitosan micelles were formulated in a drop-wise fashion of calcium chloride solution and then freeze dried for further application [133]. Emodin solution was mixed in the empty micelle solution, eventually following the same drop-wise procedure. Before determining the most efficient ratio of hydrogel to micelle, a series of analyses was conducted. First, the encapsulation efficiency and drug loading percentage was calculated using the following formulas:

(1) $$Drug\;Loading\;\%\;=\;\frac{amount\;of\;loaded\;drug}{weight\;of\;micelle\;}\times \;100,$$

(2) $$Encapsulation\;Efficiency\;\;\%\;=\;\frac{experimental\;drug\;loaded}{theoretical\;drug\;loaded\;}\times \;100.$$

The combination of hydrogel and drug-loaded micelles was developed by crosslinking sodium alginate with calcium ions to form spherical vehicles in suspended micelles. To determine the precision of the fabricated drug delivery system, the swollen and degradation ratio, and the encapsulating capacity can be calculated using the following formulas:

(3) $$Swollen\;Ratio\;=\;\frac{weight\;of\;swollen\;sample\;-\;weight\;of\;dried\;sample}{weight\;of\;dried\;sample\;}\times \;100.$$

(4) $$Degradation\;Ratio\;=\;\frac{weight\;of\;dried\;sample-\;weight\;of\;retrieved\;sample}{weight\;of\;dried\;sample\;}\times \;100,$$

(5) $$Encapsulating\;Capacity\;=\;\frac{weight\;of\;drugs\;in\;micelles}{weight\;of\;added\;drugs\;}\times \;100.$$

The release mechanism was modeled by zero and first-order equations by Higuchi and Ritger-Peppas [133]. These were used to characterize how different ratios were represented. The response surface analysis was conducted to determine how multiple factors can simultaneously affect a response. The factors investigated were calcium chloride ion, chitosan, and β-GP on the drug loading and encapsulation efficiency [133]. An abundance of calcium chloride ions increased the drug loading capacity at a restricted amount, as beyond that limit, it had a negative effect on drug loading. The excessive ions competed with phosphate attached to the surface of micelles, making the drug loading weaker [133]. Thus, it was concluded that there is a need for an appropriate concentration of calcium chloride. Overall, the factors enhanced the drug delivery system by decreasing the degradation rate, mechanical stability and flexibility, and drug release components. Nevertheless, an excess or lack of factors could negatively impact the outcome of the drug delivery system.

Cong et al. selected optimal values for the concentrations of calcium chloride ion, chitosan, and β-GP [133]. Based on the statistical analysis, the predicted values for drug loading and encapsulation efficiency were 0.68% and 86%, respectively. This was verified through a computer simulation, where the resulting values were in the proximal range as predicted. The sodium alginate hydrogels act like a barrier to improve the immediate swelling and loss of structure integrity of solely micelles. The ratios of 3:1 and 4:1 of hydrogel to micelle were observed to be a good drug delivery system with both benefits and drawbacks [133]. The swelling ratios of 3:1 and 4:1 were six and eight times greater when compared to the control over a span of a few hours. This was supported by the carboxylic groups in the alginate hydrogel responding to the external pH variations [133]. The drug release of the 3:1 ratio correlated with the Higuchi model as shown below, suggesting a slow swelling and degradation rate. Overall, it was concluded that the alginate hydrogel with chitosan micelles is a possible nanomedicine for colon-specific drug delivery therapy [133].

Tran et al. developed a method incorporating cellulose with or without chitosan combined with keratin to form a composite [130]. Ciprofloxacin was placed in the composite to study the drug delivery component. The goal was to improve the overall properties of the composite, which could in return be feasible for versatile applications [133]. Preliminary studies were conducted to understand the individual aspects of the composite. It was found that keratin, although mechanically weak, showed a slower release of ciprofloxacin than chitosan and cellulose. This property of keratin is valuable for controlling the drug release profile. The formation of the composite was through the multiple process procedure given below.

The combinations of the materials were cellulose with keratin, cellulose and chitosan with keratin, and chitosan with keratin [130]. Each combination ranged in the ratios of each component. At high concentrations of keratin, a controlled release aspect was shown. However, the opposite results were observed with either cellulose or chitosan. Therefore, it was concluded that keratin has potential in controlling drug release. The release mechanism was characterized by zero and first-order models done by Higuchi and Korsmeyer–Peppas. The first-order model resulted in an ambiguous trend, showing no correlations between the different materials in the composite. The Korsmeyer–Peppas was found to be the best-fit model for the drug release kinetics [130]. The superiority of the multicomponent composite of all three materials was demonstrated using the rate constant from the Korsmeyer–Peppas model [130]. In the chitosan or cellulose and keratin composite, the rate constant was indirectly proportional with the variation in keratin. However, when compared to the composite with all three biopolymers, the mechanical stability, controlled release, hemostasis, and bactericide were significantly better in the cellulose, chitosan, and keratin combination [130]. This novel composite could be applicable for wound healing in diabetic patients.

### 5.3. Other Applications

**Figure 9:**
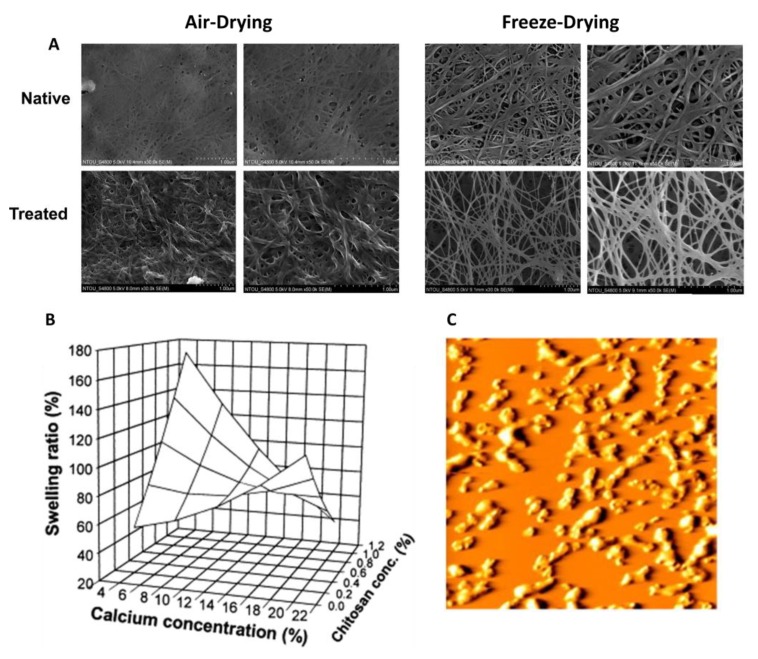
Novel applications of protein–polysaccharide composite materials: **A**) SEM images of the silymarin–zein nanoparticle/bacterial cellulose nanofibers used in a wound dressing application; **B**) Swelling ratio and release of protein from chitosan–pectin particles with change of the Ca^2+^ concentration in acidic solution; **C**) AFM 3 × 3 μm image of protein–polysaccharide (β-lactoglobulin with high methoxyl pectin) particles that were heat treated at pH 4.75, 83 °C (reproduced with permission from [114,131,144]. Copyright Elsevier, 2000, 2011, 2018).

Aside from the aforementioned applications, there are more possibilities for different protein–polysaccharide composites (Figure 9). Zhou et al. fabricated a blend of silk fibroin and cellulose acetate nanofiber through electrospinning for heavy metal ions’ adsorption [134]. This study was able to demonstrate the feasibility of the nanofibers to remove targeted metal ions through adsorption. Individually, the polysaccharides are not robust enough for such an application. When they are combined with proteins, the mechanical integrity is significantly improved. Interaction forces and shear is extensively studied to assess any improvements of the mechanical properties [135]. In addition, silk fibroin is more hydrophilic, which could be applied to metal ions found in wastewater.

Protein and polysaccharide composites are used in the food industry as mixed gels and semi-solid food products [136]. The gels have a large range of mechanical, sensorial, and textural properties [136,137]. The protein can be denatured or native and is often combined with anionic food-grade and neutral polysaccharides [138]. Whey protein is often used in the food industry along with pectin, alginate, and β-lactoglobulin [139]. Polysaccharides help control the heterogeneity of the gels and the structures of these gels can be tuned [139]. Protein–polysaccharide composites have been used in the food packaging industry [140]. These can be edible films, which have been fabricated with cellulose and whey protein to mimic biopolymers [140]. Environmental and disposal costs could be reduced with the introduction of these materials [140]. The films can possess an oil barrier, water solubility, and tastelessness [141].

In another application, hemicellulosic polysaccharides were used in a dried treatment method [142]. Wang et al. worked with protein–polysaccharide conjugates that could be stable and emulsified successfully [142]. The charged hydrophobic and hydrophilic regions aid in the emulsification, where the surface tensions are lessened, and interactions are increased. Soy hull hemicelluloses–soy protein isolate (SHH–SPI) were used in these studies, with improvements in chemical and physical properties that could ideally be used in food and pharmaceutical applications. The unique method used by these authors was the Maillard reaction. The two points in this reaction are the primary amine and the reducing end of the carbohydrate. The protein–polysaccharide composites are able to form in this reaction in a dry state without any harmful chemicals used. The conditions in this reaction, such as the relative humidity and temperature, are also controlled. Through this experiment, the SHH–SPI conjugates were prepared and characterized to fabricate an improved protein–polysaccharide composite [142], which heightened the functionality of the proteins and kept the structure the same [143].

Tsai et al. investigated the need for effective wound dressings because of the susceptibility of open wounds to infection (Figure 9A) [144]. Microbes can easily enter the body through an open wound, multiply, and cause infection. Engineered wound dressings have become popular to rapidly heal as well as prevent infections. These dressings can be manipulated to serve as a form of drug delivery for rapid healing [144]. A composite of corn zein and cellulose acetate was created to serve as a drug delivery system in wound dressings. The combination of electrospinning cellulose acetate (an acetate ester of cellulose with high hydrophilicity and high affinity for other materials) and corn zein produced an effective dressing [144]. The nanofibers produced exhibited excellent antimicrobial activity and bactericidal activity, thus resulting in less infection [144].

Jones et al. assessed the formation of biopolymer nanoparticles or microparticles with a spherical protein–ionic polysaccharide composite treated in a thermosensitive environment (Figure 9C) [29,76,114]. The biopolymer is applicable for delivery systems or food products. The goal is to provide rational design elements in forming a biopolymer particle based on the intended physicochemical and functional attributes [114]. After these studies, it was concluded that the pH, ionic strength, and thermal characteristics are critical factors in the fabrication of protein–polysaccharide-based biopolymer [114]. In addition, the physical characteristics of the biopolymer can be tuned by the temperature and time of suspension in solvent, protein-to-polysaccharide ratio, type of protein and polysaccharide combination, or co-solvent composition. Initially, the proteins attach to the polysaccharide with electrostatic attraction [114]. It was then observed that the protein was the most thermosensitive component, so the composite was heated above the denaturing temperature of the protein. The denatured protein separates from the polysaccharide due to the weakening of the electrostatic force. The separated protein mixes around in the solvent, eventually covered within the polysaccharide. As a result, the composite has a protein core and polysaccharide shell [114].

Ghosh et al. investigated the formulation of food colloids through the structures of protein–polysaccharides [145]. Food colloids are varied thermodynamic-phase systems that are covered by natural materials, such as proteins, to reduce the surface tension between the solvent. When food travels down the digestive system, it encounters a different environment pertaining to the structure, pH levels, and fluid interactions. Therefore, it is proposed that a protein–polysaccharide composite mimics the motion of food particles, releasing nutrients based on the fluid interactions and surrounding environment [114]. Protein–polysaccharide composites for such applications require controllable physicochemical properties when interacting with other liquids. The interfacial properties can support stabilization in emulsion food products. The viscoelastic and gelation properties contribute to gel-like processed food products’ ability to be unaffected by thermal treatments [145]. Similar to drug delivery applications, the active ingredients in food products can be encapsulated within the composites [145]. This application could potentially open a plethora of opportunities for advancement in health and nutrition.

## 6. Conclusions

Complexes derived from polysaccharides in combination with biodegradable proteins or proteolytic degradation of resulting products are valuable in areas, such as tissue regeneration, drug delivery, nanomedicine, health/nutrition, and water treatment. The combinations of assorted proteins extracted from various natural resources with varying polysaccharides will continue to be utilized in various applications. Depending on the targeted application, the component properties in conjunction with an appropriate fabrication technique will yield preferred fibers and biopolymers. Protein–polysaccharide complexes show promise in biomedical applications due to their versatility and affordability; however, more research is required in this area to expand upon the numerous properties that can be generated through the fabrication of such complexes.

## Figures and Tables

**Table 1:** Advantages and disadvantages of the common protein–polysaccharide composite fabrication methods.

	Advantages	Disadvantages
**Coacervation**	Easily scalable, Absence of solvents Need for common apparatus	Average diameter size is rather large Utilizes toxic cross linkers and organic solvents
**Vertical Electrospinning**	Simple Low cost equipment Can control fiber morphology Scaling capabilities	Problematic to obtain 3D structures Process depends on multiple variables Difficult to regulate the size of pores needed for biomedical applications
**Horizontal Electrospinning**	Simple Low cost equipment Can control fiber morphology Scaling capabilities	Generates average sized fibers making this less specific fabrication technique Process depends on many variables
**Phase Separation**	Morphology is easily controlled High level of consistency Simple process	Low mechanical strength High permeability of microparticles Limited to few polymers
**Cryogenic Treatment**	Longer life span of particles Less cracking failures Better electrical properties and less electrical resistance Enhanced thermal properties Improved flatness Reduced friction coefficient Easier machining	High cost Dependence on reliable source of cryogens Not suitable for very large items
